# Endobronchial valves– an iatrogenic cause of hemoptysis to be considered at autopsy

**DOI:** 10.1007/s12024-025-01009-4

**Published:** 2025-04-17

**Authors:** Erin MacDonald, Neil E. I. Langlois, Roger W. Byard

**Affiliations:** 1 Forensic Science SA, Adelaide, South Australia Australia; 2 University of Tasmania Medical School, Adelaide, Australia; 3 The Adelaide School of Biomedicine, The University of Adelaide, Level 2, Room N237, Helen Mayo North, Frome Road, Adelaide, SA 5005 Australia

**Keywords:** Chronic obstructive pulmonary disease, Emphysema, Endobronchial valve, Hemoptysis, Iatrogenic

## Abstract

An 80-year-old man who presented with hemoptysis died from ischemic heart disease and emphysema with cor pulmonale. He had a past history of ischemic heart disease with previous myocardial infarction, chronic obstructive pulmonary disease with endobronchial valve insertion and squamous cell carcinoma of the lung with lobe resection. On the day of death he had coughed up approximately one tablespoon of blood. While causes of hemoptysis usually include entities such as bronchitis, pneumonia, bronchiectasis, tumors, adjacent aneurysms, inflammatory/infective processes or septic emboli, occasionally there may be an iatrogenic etiology. The present case demonstrates a rare cause of hemoptysis associated with medical treatment - endobronchial valve insertion with surrounding granulation tissue formation and resultant hemorrhage. Hemoptysis in decedents with COPD may, therefore, be due to treatment rather than to underlying inflammatory or neoplastic lesions.

## Introduction

Hemoptysis refers to the coughing up of blood that originates from the lower respiratory tract (below the vocal cords). It may occasionally be mimicked by bleeding from the upper respiratory tract or the gastrointestinal tract. Although most often not life-threatening, a history of hemoptysis elicited at autopsy may be quite important in terms of understanding the cause and mechanism of death, as it may arise from significant respiratory disease causing death due to blood loss or asphyxiation from hemoaspiration [1–3]. Hemoptysis may also be a marker for underlying conditions ranging from acute bronchitis, pneumonia and bronchiectasis to tumors (both intrinsic and extrinsic to the airways), erosion of airways by adjacent aortic aneurysms, inflammatory/infective processes such as tuberculosis, or septic emboli in cases of infective endocarditis [4–8]. Significant hemoptysis has been defined as the expectoration of 200–600 mls of blood over 24–48 h, however, smaller volumes may also be lethal if hemoaspiration occurs [9]. In a clinical setting hemoptysis should always be taken seriously, and a history at autopsy should prompt very careful dissection and evaluation of possible underlying etiological conditions/lesions.

Hemoptysis may also arise iatrogenically from any medical procedure that injures tissues or blood vessels that abut airways such as bronchoscopy with or without biopsy, surgery, pulmonary artery catheter manipulation, or chest drain malposition. The following case is reported to demonstrate yet another possible, albeit rare, cause of iatrogenically-induced hemoptysis, that of endobronchial valve insertion.

## Case report

An 80-year-old man with a complex medical history suffered an episode of hemoptysis during which he stated that he had coughed up approximately one tablespoon of blood. He was medically assessed and found to be stable with a normal hemoglobin and an unchanged chest xray examination with no further bleeding. He was prescribed oral antibiotics with a reduced dose of his anticoagulant apixiban. Later that day he collapsed and died.

His past history included ischemic heart disease due to coronary artery atherosclerosis with a previous myocardial infarction more than 10 years previously. He had atrial fibrillation, peripheral vascular disease, hypertension, hypercholesterolemia, type II diabetes mellitus and a previous transient ischemic episode. He had undergone a remote endovascular repair of an abdominal aortic aneurysm.

In addition, he had significant respiratory disease with a right lower lobe lobectomy 10 years previously for squamous cell carcinoma (stage 2) with a presumed recurrence several years later treated by stereotactic ablative body radiotherapy (SABR). He had severe chronic obstructive pulmonary disease/emphysema with pulmonary hypertension causing marked breathlessness that limited his daily activities. He had endobronchial valves placed in bronchi of the upper lobe of the left lung for treatment of air leak with pneumothorax, with the valves being left in situ to achieve lung volume reduction.

**Fig. 1:**
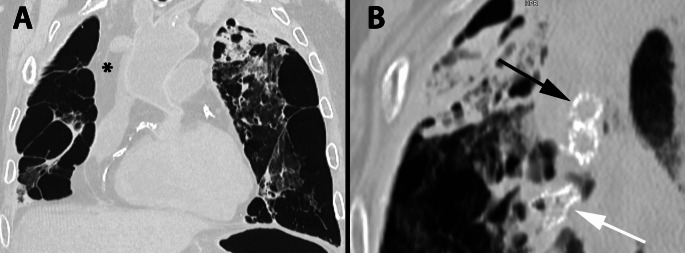
Post-mortem CT scan of the chest (lung kernal and windows; slice thickness 0.3 mm) showing severe emphysematous change and bullous transformation (most prominent on the right side post lobectomy); * marks right-sided fluid accumulation; also note calcification of the coronary arteries (**A**). Three endobronchial valves (present in the apical region of the left lung); Black arrow– valves in cross-section. White arrow– valve in longitudinal view (**B**)

Prior to autopsy, CT examination revealed marked emphysema of the lungs with numerous bullae with endobronchial valves in the bronchi of the left lung (Fig. 1). There was marked calcification of the coronary arteries. The ascending thoracic aorta and aortic arch appeared dilated with calcification of the abdominal aorta through to the iliac vessels, femoral vessels and into the arteries of the lower limbs. There was evidence of previous graft repair of the abdominal aorta, extending into the common iliac arteries.

**Fig. 2:**
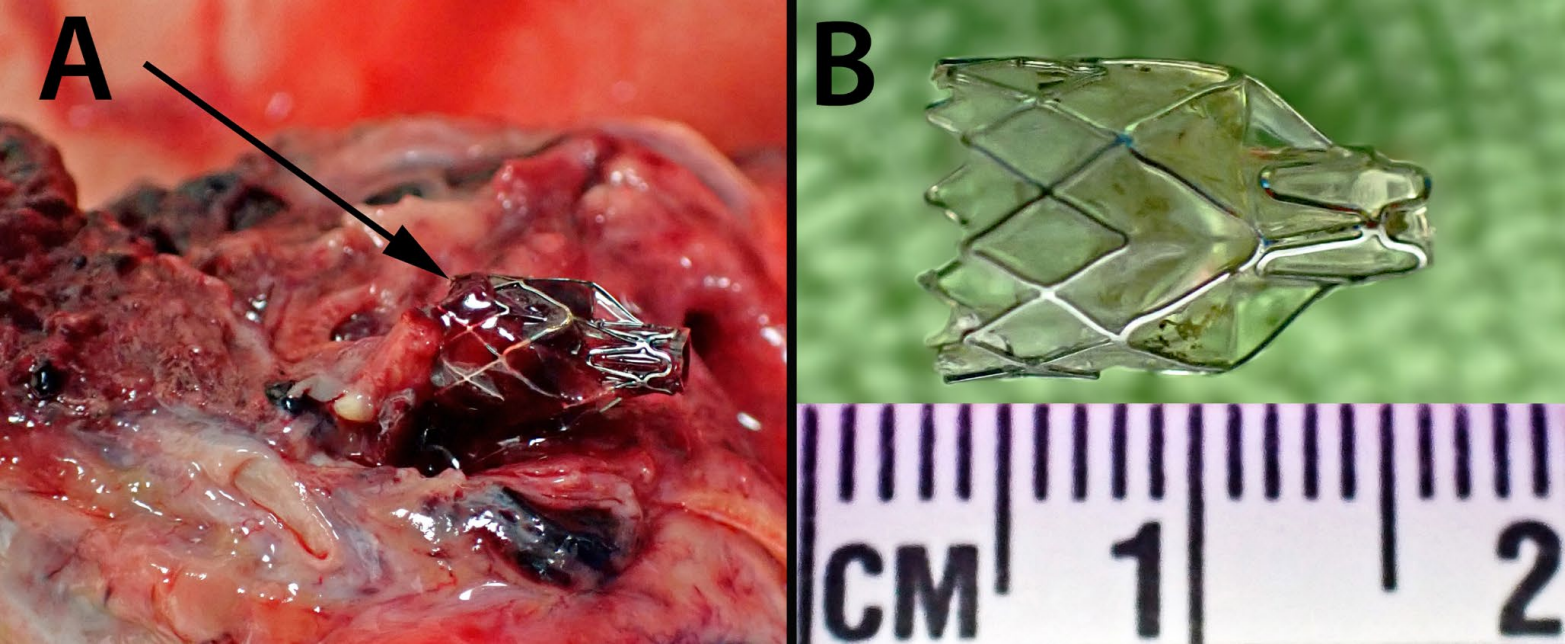
Dissection at autopsy demonstrating the endobronchial location of one of the valves (arrow) with surrounding hemorrhage (**A**). Removal of the valve revealing its structure with a wire framework (**B**)

At autopsy the presence of severe emphysema was confirmed with no evidence of tumor recurrence. Endobronchial valves were present in the upper lobe of the left lung (Fig. 2) with atelectasis of the apex of the upper lobe. There was hemorrhage around the endobronchial valves with granulation tissue formation but no bronchial wall perforation. The trachea and bronchi of the left lung were lined by blood-stained fluid. There was thickening of the pulmonary artery with right ventricular hypertrophy. The coronary arteries showed marked calcific atherosclerotic narrowing with focal myocardial fibrosis. Death was therefore attributed to ischemic heart disease and emphysema with cor pulmonale and hemoptysis.

## Discussion

Chronic obstructive pulmonary disease (COPD) is a relatively common progressive respiratory condition primarily linked to smoking. In 2022, approximately 2.5% of Australians were living with COPD which contributed to 4% of deaths that year [10]. Chronic pulmonary inflammation leads to excess mucus production, airway narrowing, and loss of alveolar structure. Over time, these processes result in chronic scarring and irreversible airway obstruction [11]. Hyperinflation, a feature of emphysema (a COPD subtype), occurs when elastin in airway walls is destroyed reducing recoil. This results in airway collapse during exhalation and air trapping distally. Additionally, destruction of alveolar septa leads to larger alveoli and increased lung volume. Lungs with increased residual volume have reduced inspiratory capacity, as they are already close to their maximum level. Larger lung volumes may also flatten the diaphragm, reducing its ability to in generate ventilatory forces [12]. These changes significantly impact upon quality of life causing dyspnea and productive coughs.

Endobronchial valves are relatively recent bronchoscopically-inserted devices that are designed to specifically treat hyperinflation resulting from emphysema. They function as one-way valves, blocking air from entering diseased tissue causing atelectasis of the emphysematous regions, allowing healthier lobes to expand and relieve pressure on the diaphragm, improving ventilation [13, 14]. Prior to the use of endobronchial valves surgical removal of hyperinflated and dysfunctional lobes had been shown to enhance ventilation [15, 16]. Endobronchial valves, however, improve spirometry-measured lung function without the need for surgery, increasing forced expiratory volume in the first second (FEV1) and decreasing residual volume [17].

The most common complication of endobronchial valves is pneumothorax, which occurs in approximately 20% of cases, although the range is 4.6–26%. The mechanism behind pneumothorax is believed to be increased negative pressure in the pleural space after atelectasis of contralateral lung lobes, leading to bullae disruption and pneumothorax. Other complications include infection-related issues such as pneumonia and more frequent COPD exacerbations [18, 19].

Failure or ineffectiveness of the valve, if not due to migration or incorrect placement, may result from disease progression with mucus or bacterial aggregation preventing valve closure. Valve migration occurs in approximately 1.9% of cases and may lead to foreign-body airway obstruction [19, 20].

As was observed in the reported case endobronchial valves may also be associated with hemoptysis, although this is uncommon and usually not medically significant. Minor hemoptysis has been reported in 1.9–5.6% of cases and is often associated with granulation tissue formation, as in the current case [18–21]. It should be recognised therefore that more recent endobronchial treatments for emphysema may be responsible for hemoptysis in decedents with COPD rather than this finding raising the spectre of potentially more sinister inflammatory or neoplastic lesions. These should, however, still be excluded at autopsy. As there may be no history of endobronchial valve insertion provided at the time of autopsy PMCT scanning provides a very useful screening tool for such devices.

## Key points

1. An 80-year-old man who presented with hemoptysis died from ischemic heart disease and emphysema with cor pulmonale.
2. He had a past history of chronic obstructive pulmonary disease with endobronchial valve insertion.
3. At autopsy lower airway bleeding had resulted from endobronchial valve insertion with surrounding granulation tissue formation.
4. Hemoptysis in decedents with COPD may, therefore, be due to treatment rather than to underlying inflammatory or neoplastic lesions.

## Data Availability

Original data has been deidentified
